# Performance of age-adjusted whole genome sequencing telomere length in idiopathic pulmonary fibrosis

**DOI:** 10.1093/ajrccm/aamag259

**Published:** 2026-06-08

**Authors:** John S Kim, Brody Receveur, David Zhang, Shwu-Fan Ma, Yong Huang, Claire McGroder, Rachel K Putman, Anna J Podolanczuk, Jerome I Rotter, Stephen S Rich, Kevin R Flaherty, Joyce S Lee, Joseph A Lasky, Ganesh Raghu, Susan Murray, Catherine Spino, Fernando J Martinez, Andrea Oh, David A Lynch, Stephen M Humphries, Ayodeji Adegunsoye, Justin M Oldham, Gary M Hunninghake, Christine Kim Garcia, Margaret Taub, Rasika A Mathias, Michael H Cho, R Graham Barr, Imre Noth, Josyf C Mychaleckyj, Ani Manichaikul, Chad A Newton

**Affiliations:** Department of Medicine, University of Virginia School of Medicine, Charlottesville, VA, United States; Department of Medicine, Columbia University Vagelos College of Physicians and Surgeons, New York, NY, United States; Department of Genome Sciences, University of Virginia School of Medicine, Charlottesville, VA, United States; Department of Medicine, Columbia University Vagelos College of Physicians and Surgeons, New York, NY, United States; Department of Medicine, University of Virginia School of Medicine, Charlottesville, VA, United States; Department of Medicine, University of Virginia School of Medicine, Charlottesville, VA, United States; Department of Medicine, Columbia University Vagelos College of Physicians and Surgeons, New York, NY, United States; Division of Pulmonary and Critical Care Medicine, Brigham and Women’s Hospital, Harvard Medical School, Boston, MA, United States; Division of Pulmonary and Critical Care Medicine, Weill Cornell Medical Center, New York, NY, United States; Institute for Translational Genomics and Population Sciences, Department of Pediatrics, The Lundquist Institute for Biomedical Innovation at Harbor, UCLA Medical Center, Torrance, CA, United States; Department of Genome Sciences, University of Virginia School of Medicine, Charlottesville, VA, United States; Department of Medicine, University of Michigan School of Medicine, Ann Arbor, MI, United States; Department of Medicine, University of Colorado, Aurora, CO, United States; Department of Medicine, Tulane University, New Orleans, LA, United States; Division of Pulmonary, Critical Care, and Sleep Medicine, University of Washington, Seattle, WA, United States; Department of Biostatistics, University of Michigan, Ann Arbor, MI, United States; Department of Biostatistics, University of Michigan, Ann Arbor, MI, United States; Department of Medicine, University of Massachusetts Chan Medical School, Worcester, MA, United States; Department of Radiology, University of California, Los Angeles Medical Center, Los Angeles, CA, United States; Department of Radiology, National Jewish Health, Denver, CO, United States; Department of Radiology, National Jewish Health, Denver, CO, United States; Section of Pulmonary and Critical Care Medicine, University of Chicago, Chicago, IL, United States; Department of Medicine, University of Michigan School of Medicine, Ann Arbor, MI, United States; Division of Pulmonary and Critical Care Medicine, Brigham and Women’s Hospital, Harvard Medical School, Boston, MA, United States; Department of Medicine, Columbia University Vagelos College of Physicians and Surgeons, New York, NY, United States; Department of Biostatistics, Johns Hopkins University, Bloomberg School of Public Health, Baltimore, MD, United States; Genomics and Precision Health Section, National Institute of Allergy and Infectious Diseases, National Institutes of Health, Bethesda, MD, United States; Channing Division of Network Medicine, Brigham and Women’s Hospital, Harvard Medical School, Boston, MA, United States; Department of Medicine, Columbia University Vagelos College of Physicians and Surgeons, New York, NY, United States; Department of Epidemiology, Mailman School of Public Health, New York, NY, United States; Department of Medicine, University of Virginia School of Medicine, Charlottesville, VA, United States; Department of Genome Sciences, University of Virginia School of Medicine, Charlottesville, VA, United States; Department of Genome Sciences, University of Virginia School of Medicine, Charlottesville, VA, United States; Division of Pulmonary and Critical Care Medicine, University of Texas Southwestern Medical Center, Dallas, TX, United States

**Keywords:** telomere length, pulmonary fibrosis, whole genome sequencing

## Abstract

**Rationale:**

Whole genome sequencing–estimated telomere length (WGS-TL) correlates with directly measured peripheral blood telomere length. However, raw WGS-TL does not account for normal age-related telomere attrition to identify patients with idiopathic pulmonary fibrosis (IPF) with pathologically short telomeres.

**Objectives:**

To determine utility of age-adjusted WGS-TL to identify adults with telomere-related qualifying variants (TRQVs) and whether it associates with survival.

**Methods:**

WGS-TL from two samples of community-dwelling adults (MESA) and people who smoke cigarettes (COPDGene) were used to construct an age-adjusted WGS-TL nomogram (*n* = 9940) using linear regression models that regressed on raw WGS-TL and adjusted for chronologic age. Using this nomogram, age-adjusted WGS-TL was generated in IPF cases from the Pulmonary Fibrosis Foundation Patient Registry (PFF-PR) (*n* = 825) to determine diagnostic performance characteristics for identifying TRQV and survival discrimination.

**Measurements and Main Results:**

There was a higher proportion of IPF patients from the PFF-PR with abnormally short age-adjusted WGS-TL <10th percentile (28%) compared with MESA (5%) or COPDGene (10%). Among IPF patients, age-adjusted WGS-TL <10th percentile outperformed raw WGS-TL <10th percentile at discriminating TRQV (area under the receiver operating curve = 0.66 [95% CI, 0.60-0.73] vs 0.62 [95% CI, 0.56-0.69]). At both the shorter age-adjusted and raw WGS-TL thresholds, specificity (0.74-0.99) was higher than sensitivity (0.10-0.58). Age-adjusted WGS-TL below the 10th percentile was associated with shorter 3-year transplant-free survival (HR, 1.35 [95% CI, 1.05-1.74]) but did not improve survival discrimination over clinical variables alone.

**Conclusions:**

Age-adjusted WGS-TL enables identification of IPF patients with pathologically short telomeres, modestly improves detection of TRQV, and associates with survival.

At a Glance Commentary **Current Scientific Knowledge on the Subject:** Raw whole genome sequencing–estimated telomere length (WGS-TL) correlates weakly with survival and modestly with telomerase mutation status in idiopathic pulmonary fibrosis.

**What This Study Adds to the Field:** After accounting for age, shorter WGS-TL associates with shorter transplant-free survival and telomerase mutation status.

## Introduction

Idiopathic pulmonary fibrosis (IPF) is characterized by recurrent injury to the lung that can lead to fibrosis. Treatments are limited in their scope and do not reverse fibrosis.^1^^,^^2^ Prior genetic studies have identified rare variants in telomere-maintenance genes that not only predispose to IPF development but also confer substantial mortality and progression risk.^3-7^ While telomere length normally shortens with age, these damaging variants result in pathologic shortening of the telomere length, which itself is associated with rapid lung function decline and poor survival in pulmonary fibrosis.^8-10^ Furthermore, telomere length may be a useful marker to inform treatment strategies in non-IPF interstitial lung disease (ILD) subtypes.^11-13^

The methods to directly measure telomere length include quantitative polymerase chain reaction (qPCR), terminal restriction fragment analysis by Southern blot, and fluorescence in situ hybridization coupled with flow cytometry (flow-FISH).^4^^,^^5^^,^^8^^,^^14-16^ Given normal telomere dynamics, nondiseased control populations have been assembled to derive age-adjusted telomere length values to quantify pathologic telomere shortening and understand the contribution of telomere dysfunction to disease. Recent bioinformatic pipelines have been developed to estimate telomere lengths from whole genome sequencing (WGS), which correlate with laboratory-based telomere measurements^17^ but are associated with less precise prognostic effect^18^ compared to age-adjusted direct measurements.^13^ However, the inability to differentiate normal age-related telomere attrition from pathologic telomere shortening impedes the potential diagnostic and prognostic utility of estimating telomere length from WGS. Existing WGS-TL studies typically incorporate age as a covariate in regression models or analyze within disease cohorts, rather than anchoring individual WGS-TL estimates to an external, population-based age reference. As a result, WGS-derived telomere length remains difficult to interpret across studies and cohorts, limiting its utility for variant prioritization, cross-cohort harmonization, and population-based analyses of telomere biology.

To address this gap, we leveraged the WGS-estimated telomere length (WGS-TL) obtained from two large US adult cohorts without radiologic evidence of pulmonary fibrosis to construct an external, population-based age-adjusted WGS-TL reference nomogram. This approach enables standardized interpretation of WGS-TL across independent cohorts, rather than relying on within-cohort normalization or regression-based age adjustment. We then compared raw and age-adjusted WGS-TL diagnostic and prognostic discrimination within a multicenter prospective IPF registry. We hypothesized that age-adjusted WGS-TL would outperform raw WGS-TL for discriminating presence of telomere-gene qualifying variants and three-year transplant-free survival. While WGS-TL is not a clinically scalable assay, this framework is directly applicable to large epidemiologic and gene discovery studies where WGS data already exist, providing a consistent method to contextualize telomere length and prioritize telomere biology across datasets.

## Methods

### Study sample

We used data from baseline visits of the Multi-Ethnic Study of Atherosclerosis (MESA) and Genetic Epidemiology of Chronic Obstructive Pulmonary Disease (COPDGene) cohorts within the Trans-Omics for Precision Medicine (TOPMed) program to generate a nomogram of WGS-TL versus age.^19^ MESA is an NHLBI-funded cohort of community-dwelling adults from six US communities who were recruited at Exam 1 (2000-2002) between the ages of 45 and 84 years and without known cardiovascular disease.^20^ COPDGene is a case-control study that recruited adults the ages of 45 to 80 years with ≥10-cigarette pack-year history during Phase I (2008-2011). Both MESA and COPDGene participants had interstitial lung abnormalities (ILAs) adjudicated from their Exam 1 and Phase 1 computed tomography (CT) scans, respectively.^21^^,^^22^ Subjects without evidence of fibrotic ILA on baseline scans were included in the nomogram construction. Because cigarette smoking is a common risk factor for IPF, this was the rationale to also use the COPDGene cohort to derive the nomogram.^23^

To examine associations of raw and age-adjusted WGS-TL with survival in IPF, we used clinical and WGS data from the Pulmonary Fibrosis Foundation Patient Registry (PFF-PR).^24^ Patients with a clinical diagnosis of ILD evaluated within the PFF Care Center Network were enrolled in the registry and provided blood samples. PFF-PR patients with a diagnosis of IPF and who had WGS-TL were included in this analysis. WGS data were acquired through the Database of Genotypes and Phenotypes (dbGaP) for MESA (phs001416.v1.p1) and COPDGene (phs000951). All subjects for PFF-PR provided informed consent, and institutional review board approval was obtained for this study (study number 21787).

### WGS-TL measurements

Whole blood DNA samples were collected and stored at Exam 1 in MESA and Phase I in COPDGene. WGS was performed on these samples using Illumina HiSeq X Ten instruments and targeted a mean depth of at least 30× (paired-end, 150-bp reads).^19^ KAPA Biosystems PCR-free library preparation kits were used for all of the sequencing. Telomere length was obtained from WGS using TelSeq as previously described.^17^ Counts of sequencing reads that contained fixed number of repeats of the telomeric nucleotide motif, TTAGGG, were used in the estimation of telomere lengths. Due to possible technical sources of variation in telomere length estimates within and across MESA and COPDGene, batch-adjusted telomere length estimates were used to construct the age-adjusted nomogram as described previously.^17^ The batch-adjusted WGS-TL estimates were generated from several TOPMed-related cohorts, which included MESA and COPDGene, and attempted to account for technical confounders both within and across study cohorts. The overall mean WGS-TL was added to the batch-adjusted WGS-TL to put it on a measured scale. For the PFF-PR, WGS was performed from whole blood DNA samples in a single batch at Psomagen (Rockville, MD, USA) as previously described.^25^ TelSeq version 0.02 was utilized to extract telomere length in kilo-base pairs (kb) generated from WGS data mapped to hg38 at a mean read depth of 36.2 (range, 27.9-109.5).^26^ DRAGEN version 3.10.4 (Illumina) was used for mapping. Motif setting of 12 TTAGGG/CCCTAA repeats in TelSeq were used for the read length.^27^

We defined rare telomere-related gene qualifying variants (TRQVs) using an approach previously described^28^ and restricted to single-nucleotide and insertion-deletion variants in the following genes: *TERT*, *RTEL1*, *PARN*, *TINF2*, *DKC1*, *TERC*, and *NAF1*. Protein-truncating variants, including frameshifts, stop-gain, start-loss, and splice site variants, were differentiated from missense variants. IPF cases from the PFF-PR with multiple gene mutations were categorized as TRQV if at least one was within a telomere-related gene.

### Statistical analysis plan

A linear regression model of WGS-TL versus age was used to create a nomogram, similar to prior studies.^6^^,^^29^^,^^30^ As MESA is more representative of a general population, and all COPDGene participants had a history of smoking, we combined these cohorts to both improve statistical power and generalizability given that nearly 70% of patients with IPF have a history of cigarette smoking.^2^^,^^31^^,^^32^ To validate the regression models, we tested standard assumptions, including linearity, normality, independence of errors, and homoscedasticity, using diagnostic plots. Aware that outliers may influence the nomogram, we defined an individual as an outlier if there were residuals exceeding 5 standard deviations (SD) from the mean. There were 2 significant outliers in COPDGene that were detected in the linear regression models. These subjects were removed from the combined cohort as to not significantly influence the model’s slope. We then constructed nomograms from the combined MESA and COPDGene cohorts using the linear regression model output to derive age-adjusted WGS-TL thresholds at the 1st, 10th, and 50th percentiles. Because linear regression models of telomere length versus age have been used to generate nomograms in prior pulmonary fibrosis studies,^6^^,^^29^ we used a locally estimated scatterplot smoothing (LOESS) plot to assess linearity between chronological age and WGS-TL in the MESA and COPDGene participants and determine whether there was an age cutoff in which linearity was reduced. If so, we would restrict the cohort to generate the nomogram based on that age cutoff to ensure linearity. LOESS plot revealed curvature in telomere length trends for individuals >80 years of age (Figure S1). Given this deviation, participants >80 years were excluded from the model. We then constructed a nomogram based on the linear model output (expected age-adjusted WGS-TL = 4.432 − 0.0194 × age), to derive the median expected WGS-estimated TL, representing the 50th percentile, along with the expected 1st and 10th percentile thresholds for each age. The age-adjusted WGS-TL values were calculated by subtracting the raw WGS-TL from the expected WGS-estimated TL values for PFF-PR participants and categorized according to the age-adjusted WGS-estimated TL thresholds. Because nearly 80% of patients^8^ with telomere gene variants have age-adjusted TL <10th percentile using direct measurements and the 10th percentile threshold has been consistently associated with differential mortality and progression risk, we chose this age-adjusted WGS-TL threshold as our primary analysis.^4-6^^,^^18^

To quantify the overall diagnostic performance, we stratified patients by presence of TRQV and age-adjusted WGS-TL 1st, 10th, and 50th percentile thresholds. We also stratified patients by corresponding raw WGS-TL 1st, 10th, and 50th percentiles using within-cohort quantiles (ie, shortest 10% of raw WGS-TL vs longest 90% of raw WGS-TL). Two-by-two tables were constructed with PFF-PR patients stratified by either age-adjusted WGS-TL percentile or raw WGS-TL quantile versus presence of TRQV to calculate discrete test performance characteristics (sensitivity, specificity, positive predictive value [PPV], and negative predictive value [NPV]). Overall discriminative performance was then quantified by the area under the receiver operating curve (AUROC).

To assess prognostic performance, we examined associations of raw and age-adjusted WGS-TL with three-year lung transplant free survival (TFS) in the PFF-PR, whereby transplant and death were considered equal. Patients were categorized using the same age-adjusted and raw WGS-TL thresholds as outlined above. Multivariable Cox proportional hazards models were used to assess the associations of continuous and categorical raw and age-adjusted WGS-estimated telomere length on 3-year TFS. The models were adjusted for clinical covariates including gender-age-physiology (GAP) stage,^33^ antifibrotic exposure at time of enrollment, self-reported smoking history (never vs ever) and first 10 principal components of ancestry. Patients with missing baseline percent predicted forced vital capacity (FVC) and diffusing capacity of the lung for carbon monoxide were excluded from this analysis. Harrell’s concordance index (C-index) was used to assess overall mortality risk discrimination.^33^^,^^34^ Two-sided *P* values <.05 were considered statistically significant. R version 4.4.1 (R Foundation for Statistical Computing) software was used for the analyses.

## Results

There were 4068 and 5872 participants in MESA and COPDGene, respectively, with WGS-TL and without radiological evidence of lung fibrosis (Figure 1). There were 825 PFF-PR patients with a clinical diagnosis of IPF and WGS-TL measurements. There was a higher proportion of females and lower proportions of non-Hispanic White individuals in MESA than in COPDGene and PFF-PR (Table 1). There were more people who smoke with higher pack-year history in COPDGene than in MESA or PFF-PR. The mean raw WGS-TL was shorter in the PFF-PR cohort (2.60 kb [SD, 0.74]) than either MESA (3.37 kb [SD, 0.70], *P* = 2.2 × 10^−16^) or COPDGene (3.21 kb [SD, 0.67], *P* = 2.2 × 10^−16^). Within the PFF-PR cohort, raw WGS-TL was weakly correlated with chronological age (*r = *−0.14, *P* = 5.1 × 10^−6^) but not with FVC (*r* = −0.04, *P* = .26). There were no differences in raw WGS-TL with respect to demographic or IPF-specific features (Figure S2).

**Figure 1:**
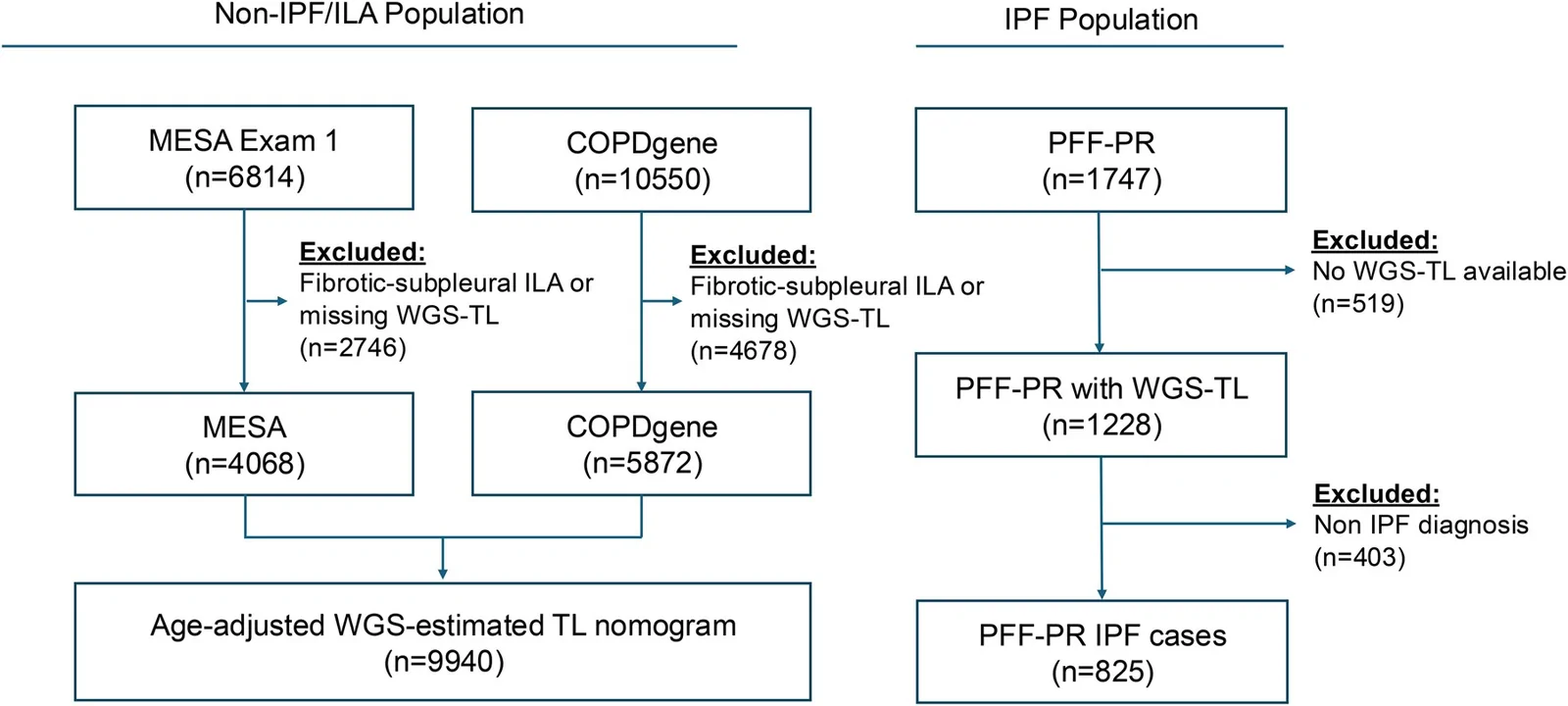
STrengthening the Reporting of OBservational studies in Epidemiology (STROBE) diagram. Abbreviations: COPDGene, Genetic Epidemiology of Chronic Obstructive Pulmonary Disease; ILA, interstitial lung abnormality; IPF, idiopathic pulmonary fibrosis; MESA, Multi-Ethnic Study of Atherosclerosis; PFF-PR, Pulmonary Fibrosis Foundation Patient Registry; TL, telomere length; WGS-TL, whole genome sequencing–estimated telomere length.

**Table 1:** Baseline characteristics.

Characteristic	MESA	COPDGene	PFF-PR (*n* = 825)
	(*n* = 4068)	(*n* = 5872)	
**Age, y, mean (SD)**	61 (10)	59 (9)	71 (8)
**Female sex, No. (%)**	2095 (51.5%)	2710 (46.2%)	203 (25%)
**Self-reported race and ethnicity, No. (%)**			
** Non-Hispanic White**	1683 (41.4%)	4008 (68.3%)	748 (93%)
** African American or Black**	980 (24.1%)	1864 (31.7%)	7 (1%)
** Hispanic**	869 (21.4%)	0	29 (4%)
** Asian**	536 (13.2%)	0	20 (2%)
**Ever smoked, No. (%)**	2011 (49.4%)	5812 (99.0%)	538 (65%)
**Cigarette pack-years, mean (SD)**	10.8 (20.6)	43.0 (23.5)	13.4 (15.3)
**WGS-TL, kb, mean (SD)**	3.37 (0.70)	3.21 (0.67)	2.60 (0.74)

Abbreviations: COPDGene, Chronic Obstructive Pulmonary Disease Genetic Epidemiology; MESA, Multi-Ethnic Study of Atherosclerosis; PFF, Pulmonary Fibrosis Foundation Patient Registry; SD, standard deviation; WGS-TL, whole genome sequencing–estimated telomere length.

### Age-adjusted WGS-TL

The age-adjusted WGS-TL nomogram was constructed from the combined MESA and COPDGene cohorts after excluding outliers (*n* = 2) and subjects >80 years old (*n* = 117) and applied to the PFF-PR IPF cohort (Figure 2 and Supplementary File). There were 47.2% of participants in the MESA and 58.1% in the COPDGene cohort that had shorter than expected telomere length after adjusting for age (Figure 3). In contrast, 81% (*n* = 667) of the PFF-PR cohort had age-adjusted WGS-TL below the 50th percentile. This trend continued for shorter age-adjusted telomere length thresholds whereby 5.3% of MESA, 10.6% of COPDGene, but 28% (*n* = 233) of the PFF-PR IPF cohort fell below the 10th percentile threshold. Although PFF-PR patients with age-adjusted WGS-TL <10th percentile were younger and had a higher proportion of familial disease than those with WGS-TL ≥10th percentile, no other demographic or IPF-specific feature differentiated these patients (Table S1).

**Figure 2:**
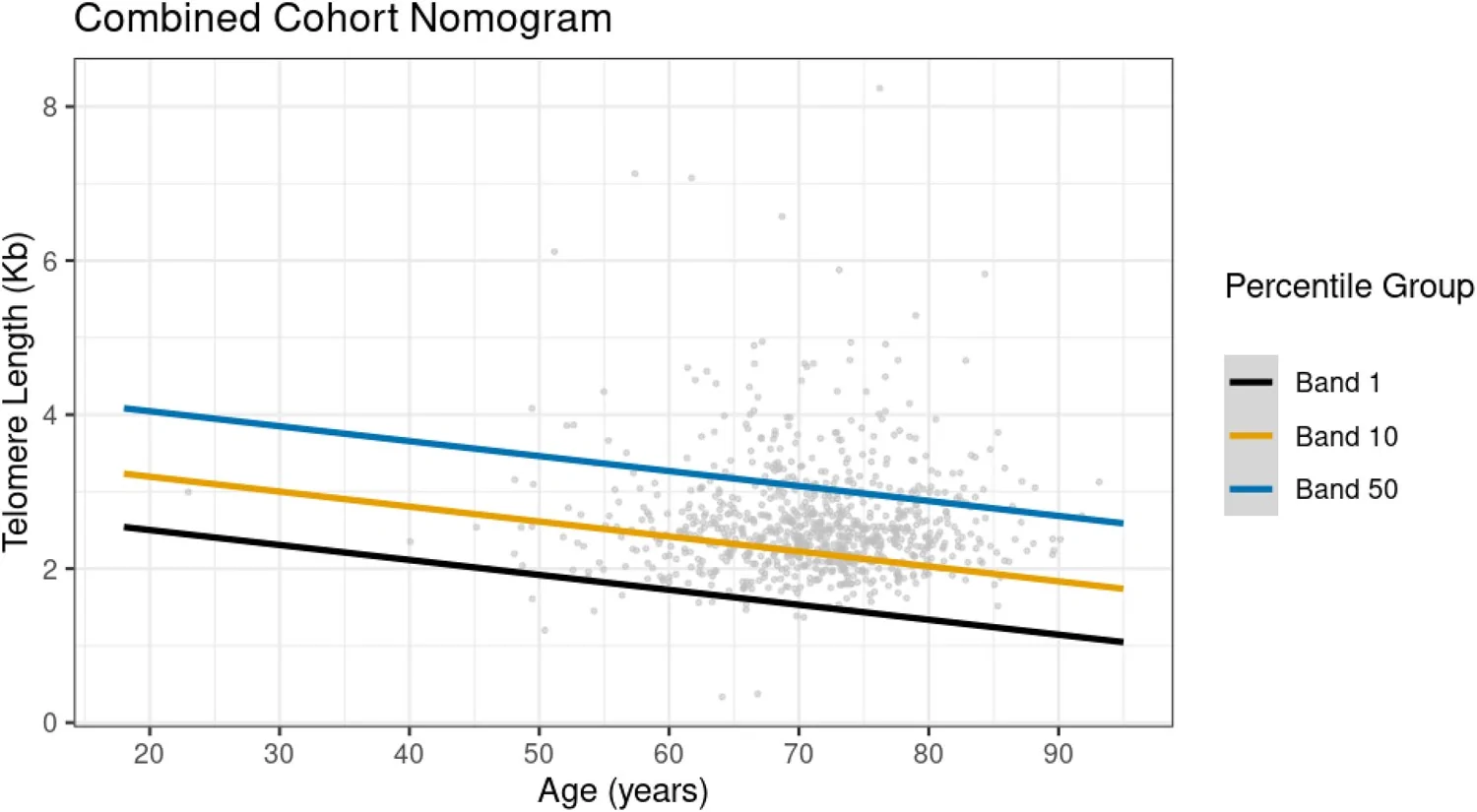
Age-adjusted whole genome sequencing–based telomere length nomogram constructed from 9821 participants from the Multi-Ethnic Study of Atherosclerosis (MESA) and Genetic Epidemiology of Chronic Obstructive Pulmonary Disease (COPDGene) cohorts and applied to Pulmonary Fibrosis Foundation Patient Registry idiopathic pulmonary fibrosis patients (gray dots).

### Telomere-related gene qualifying variants

Within the PFF-PR, there were 60 patients (7%) who harbored qualifying variants (QVs) with telomere-maintenance genes, of which 25 (42%) were protein truncating or splice variants and 35 (58%) were missense variants (Table S2). Most QVs were in *TERT* (*n* = 20 [33%]) followed by *RTEL1* (*n* = 15 [25%]) and *PARN* (*n* = 12 [20%]). Patients harboring a TRQV had shorter raw and age-adjusted WGS-TL compared with non-telomere gene QVs (*ABCA3*, *KIF15*, and *SPDL1*) and noncarriers. Telomere-maintenance gene QVs also occurred more frequently in patients with familial pulmonary fibrosis (*n* = 25 [14%]) compared to sporadic IPF (*n* = 35 [5%], *P* = 8.7 × 10^−5^).

**Table 2:** Diagnostic performance characteristics identifying telomere-related gene qualifying variants using age-adjusted and raw WGS-TL thresholds in the Pulmonary Fibrosis Foundation Patient Registry idiopathic pulmonary fibrosis cohort (*n* = 825).

Characteristic	Sensitivity	Specificity	PPV^a^	NPV^a^	AUROC (95% CI)
**Family history**	0.42	0.81	0.14	0.95	0.61 (0.55-68)
**Age-adjusted WGS-TL <50th pct**	0.98	0.21	0.09	0.99	0.59 (0.57-0.62)
**Raw WGS-TL <50th pct**	0.78	0.46	0.10	0.96	0.62 (0.57-0.68)
**Age-adjusted WGS-TL <10th pct**	0.58	0.74	0.15	0.96	0.66 (0.60-0.73)
**Raw WGS-TL <10th pct**	0.35	0.90	0.21	0.95	0.62 (0.56-0.69)
**Age-adjusted WGS-TL <1st pct**	0.13	0.99	0.62	0.94	0.56 (0.52-0.61)
**Raw WGS-TL <1st pct**	0.10	0.99	0.67	0.93	0.55(0.51-0.59)

Abbreviations: AUROC, area under the receiver operating characteristic curve; CI, confidence interval; NPV, negative predictive value; pct, percentile; PPV, positive predictive value; WGS-TL, whole genome sequencing estimated telomere length.

a Prevalence of telomere-gene qualifying variant = 7%.

In the PPF-PR cohort, IPF patients harboring a TRQV and those with familial pulmonary fibrosis were younger than noncarriers and those with sporadic disease (Figure S3). Because of the known age–telomere length relationship, we then binned patients by age to mitigate its effect and examined the association of raw and age-adjusted WGS-TL across family history and variant strata. Among IPF patients, raw WGS-TL did not differ across age groups, but age-adjusted WGS-TL increased with advancing age (Figure S4), suggesting that adjusting WGS-TL for age more effectively captured pathologic telomere shortening associated with earlier age of IPF onset. This trend was maintained after restricting to IPF patients harboring TRQV and IPF patients with a family history of pulmonary fibrosis.

**Figure 3:**
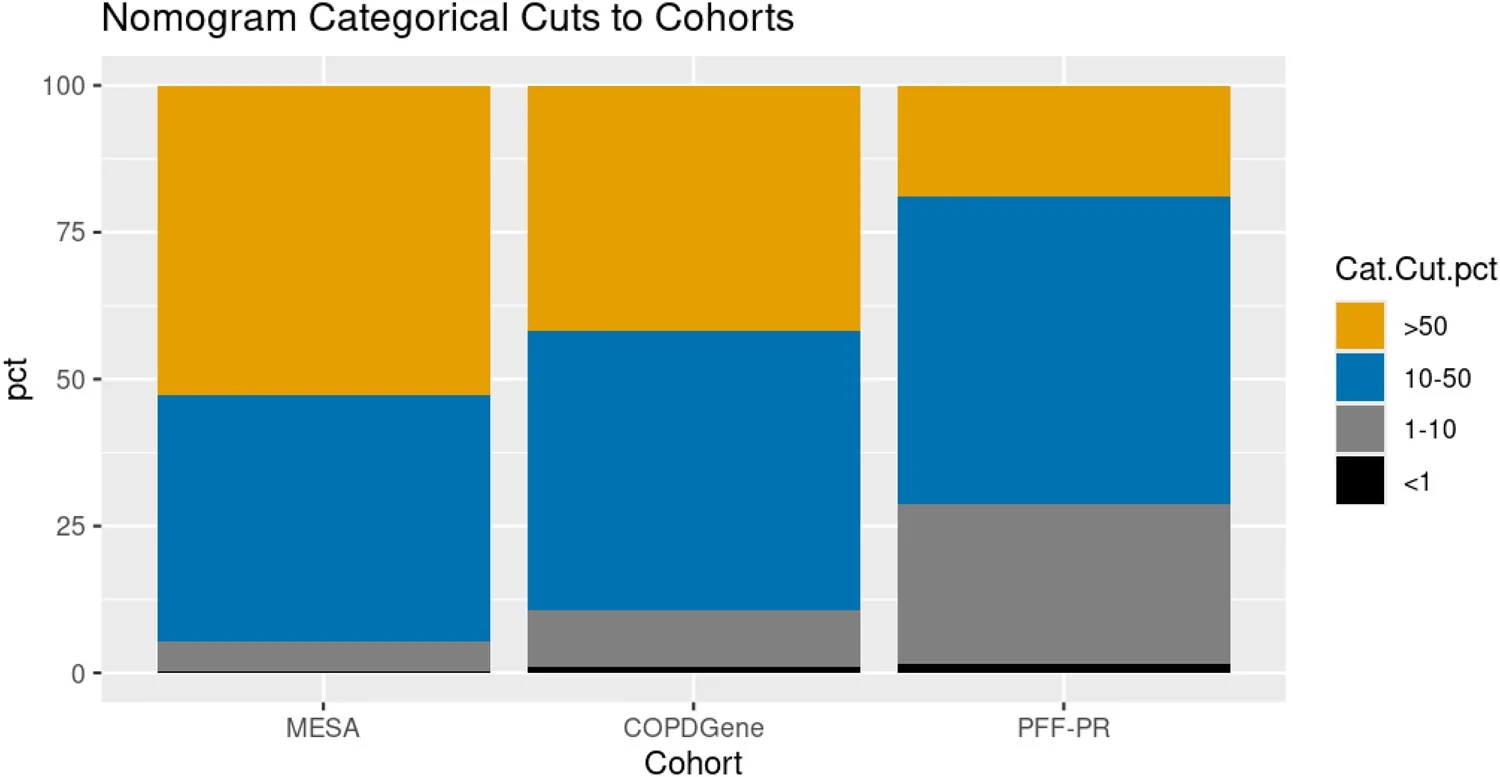
Stacked barplot for age-adjusted whole genome sequencing–estimated telomere length categories (<1st percentile, 1-10th percentile, 10-50th percentile, >50th percentile) for the Multi-Ethnic Study of Atherosclerosis (MESA), Genetic Epidemiology of Chronic Obstructive Pulmonary Disease (COPDGene), and Pulmonary Fibrosis Foundation Patient Registry (PFF-PR) cohorts.

### Age-adjusted WGS-TL diagnostic performance

Age-adjusted WGS-TL as a continuous variable provided better discrimination for the presence of telomere gene QV (AUROC, 0.75 [95% CI, 0.68–0.81]) compared to raw WGS-TL (AUROC, 0.71 [95% CI, 0.64–0.78]). The age-adjusted 10th percentile threshold outperformed the other thresholds but offered only modest discrimination for predicting telomere gene QV status (AUROC 0.66 [95% CI, 0.60–0.73]) (Table 2). Specificity was higher than sensitivity for the shorter raw and age-adjusted WGS-TL thresholds. The age-adjusted WGS-TL 50th percentile threshold had sensitivity of 0.98 but specificity of only 0.21 given the high false-positive rate. At the other extreme, the age adjusted WGS-TL 1st percentile threshold produced specificity of 0.99 and sensitivity of 0.13 because of a high false-negative rate. NPV was high across all raw and age-adjusted WGS-TL thresholds (0.94-0.99) but PPV was low (0.08-0.62) given the low prevalence of the telomere gene QV at 7%. Similar trends were also observed in the sensitivity analysis restricting telomere-gene QVs to protein-truncating variants but with attenuated overall discrimination (Table S3). However, among IPF patients with a family history of PF, the diagnostic performance of age-adjusted WGS-TL <10th percentile improved with an AUROC of 0.75 (95% CI, 0.67-0.84) (Table S4).

**Table 3:** Three-year transplant-free survival association and discrimination for age-adjusted and raw WGS-TL thresholds (*n* = 755).

Models^a^	Hazard ratio (95% CI)	*P* value	Harrell’s C-index
**Clinical model**			0.709 (0.681-0.738)
**Clinical model + TRQV**	1.83 (1.25-2.69)	0.002	0.714 (0.687-0.742)
**Clinical model + age-adjusted WGS-TL (continuous)**	0.82 (0.69-0.98)	0.033	0.712 (0.684-0.739)
**Clinical model + raw WGS-TL (continuous)**	0.87 (0.73-1.04)	0.12	0.711 (0.683-0.739)
**Clinical model + age-adjusted WGS-TL <50th pct**	1.32 (0.95-1.82)	0.09	0.710 (0.682-0.738)
**Clinical model + raw WGS-TL <50th pct**	1.19 (0.94-1.51)	0.14	0.710 (0.682-0.738)
**Clinical model + age-adjusted WGS-TL <10th pct**	1.38 (1.07-1.78)	0.012	0.713 (0.685-0.741)
**Clinical model + raw WGS-TL <10th pct**	1.38 (0.98-1.94)	0.07	0.710 (0.682-0.739)
**Clinical model + age-adjusted WGS-TL <1st pct**	1.70 (0.75-3.86)	0.20	0.710 (0.682-0.738)
**Clinical model + raw WGS-TL <1st pct**	2.07 (0.84-5.10)	0.11	0.711 (0.682-0.739)

Abbreviations: CI, confidence interval; pct, percentile; TRQV, telomere-related gene qualifying variant; WGS-TL, whole genome sequencing–estimated telomere length.

a Clinical model includes gender-age-physiology (GAP) stage, smoking history, antifibrotic exposure at enrollment, and first 10 principal components. Excluded patients with missing GAP score (*n* = 70).

### Age-adjusted WGS-TL prognostic performance

In the PFF-PR, shorter continuous age-adjusted WGS-TL, but not raw WGS-TL, was associated with worse three-year transplant free survival (Table 3). Among the evaluated dichotomous thresholds, only age-adjusted WGS-TL <10th percentile was independently associated with worse survival (HR, 1.38 [95% CI, 1.07-1.78], *P* = .012) after accounting for clinical covariates. Even after excluding patients with a self-reported family history of pulmonary fibrosis, WGS-TL <10th percentile remained associated with shorter survival (HR, 1.30 [95% CI, 1.01-1.67]) (Table S5). Antifibrotic use did not modify the association between age-adjusted WGS-TL and survival (*P* value for interaction = .86). WGS-TL <10th percentile was more strongly associated with shorter survival among patients younger than 65 years compared with older patients (*P* value for interaction = .04) (Table S6). The presence of a telomere gene QV was also associated with higher mortality risk independent of the clinical covariates (HR, 1.83 [95% CI, 1.25-2.69], *P* = .002). However, despite significant survival associations, neither WGS-TL measurement nor TRQV status substantially improved prognostic discrimination over clinical variables alone (Table 3).

## Discussion

In this multicenter study, we created an age-adjusted WGS-TL nomogram from adults without ILD and applied it to IPF patients and evaluate the ability of WGS-TL to identify those with pathologically short telomeres. Because direct TL measurements using validated clinical assays (qPCR or flow-FISH) were not available, our findings should be interpreted as methodological advances in WGS-TL interpretation rather than validation against a clinical reference standard. We found that age-adjusted WGS-TL identified IPF patients with shorter telomeres than expected for age, which corresponded to younger IPF onset, mirroring prior pulmonary fibrosis studies.^4^^,^^7^ Age-adjusted WGS-TL outperformed raw WGS-TL in identifying TRQV, with the best performance observed in patients with a family history of pulmonary fibrosis. Age-adjusted WGS-TL <10th percentile was associated with higher mortality risk, but neither this threshold nor any other WGS-TL variable improved survival discrimination over readily available clinical variables. These data suggest that our age-adjustment WGS-TL nomogram differentiated normal age-related telomere attrition from pathologic telomere shortening and could inform future studies that seek to leverage this technology.

Unlike other static genetic biomarkers, telomere length normally shortens with advancing age. Therefore, clinicians and researchers have accounted for age when quantifying pathologic telomere shortening that drives short telomere syndrome manifestations.^6^ For direct telomere measurement methods, healthy control populations have been assembled to construct age-adjusted nomograms and quantify pathologic attrition.^5^^,^^6^^,^^8^ To our knowledge, this is the first study to generate age-adjusted WGS-TL estimates in a large cohort of adults without radiologic evidence of ILD. We found that the majority of IPF cases had WGS-TL shorter than expected for their age, and 28% had age-adjusted WGS-TL below the 10th percentile, similar to the prevalence of short telomeres reported in other IPF cohorts using direct measurement methods.^5^^,^^6^^,^^8^^,^^35^ By generating expected telomere length using >9000 individuals spanning the adult age spectrum (Supplemental File), we provide a reference framework that may enhance cross-study comparability and facilitate more standardized interpretation of WGS-TL across cohorts. In contrast, the common practice of within-cohort categorization produces artificial thresholds that are specific to the study sample and often difficult to transport to external cohorts. However, application across independently sequenced cohorts may still require cohort-specific calibration to account for technical differences.

Age-adjusted WGS-TL in IPF, but not raw WGS-TL, was associated with worse survival, and reinforces the notion that biologically abnormal telomere attrition better explains the telomere–survival relationship in IPF. This observation is consistent with prior studies in which raw WGS-TL was not associated with mortality and extends those findings by contextualizing that WGS-TL relative to age reveals a prognostically relevant signal.^18^ However, unlike prior work using qPCR-based age-adjusted TL that modestly improved survival discrimination,^8^ we found that neither raw nor age-adjusted WGS-TL improved survival prediction beyond standard clinical variables. This should be interpreted in light of the known limitations of the C-index, which is relatively insensitive to incremental biomarkers added to models that already include strong clinical predictors and may underestimate improvements in risk stratification.^36^ This discrepancy may be due to multiple factors including cohort composition and availability of antifibrotic therapies, but also underscores an important limitation of the present study as WGS-TL was not directly validated against laboratory-based telomere measurements in the same individuals. This also suggests that WGS-derived telomere length estimates, while scalable and reproducible, may have lower accuracy than directly measured telomere length.^18^

While the high cost and complexity of WGS interpretation limit its clinical applicability, WGS is increasingly being used for gene discovery in IPF and other telomere-related diseases. Our findings provide a framework for leveraging age-adjusted WGS-TL as a functional metric to qualify potentially damaging variants in telomere-maintenance genes. The proportion of patients with TRQV in other IPF studies has been reported to be 4% to 13%, and the prevalence of 7% in this study falls within that range.^25^^,^^37^ We found that WGS-TL yields high specificity but poor sensitivity in identifying individuals with TRQV, suggesting that the absence of short age-adjusted WGS-TL may be more informative for ruling out a telomere gene variant of interest, whereas presence of short age-adjusted WGS-TL may be insufficient to confirm variant pathogenicity and would require functional validation. Therefore, integrating age-adjusted WGS-TL into gene discovery pipelines could enhance qualification of telomere gene variants at a population level.

There are several limitations of this study. Most notably, we did not have paired direct telomere length measurements in the PFF-PR cohort, precluding direct validation of WGS-TL against established clinical reference standards. As a result, we used telomere gene QVs as a pragmatic comparator to assess the relative performance of age-adjusted WGS-TL versus raw WGS-TL values, recognizing that variant pathogenicity itself may be uncertain. Future studies incorporating paired WGS-TL and direct telomere measurements from the same individuals will be essential to fully define the accuracy and limitations of WGS-derived estimates. Although our age-adjustment nomogram was developed in large, ethnically diverse cohorts, our application to the PFF-PR was largely limited to older, White patients and thus the generalizability of age-adjusted WGS-TL to other groups is unknown. This is relevant due to studies that suggest differences in clinical outcomes by self-reported race, although shorter telomere length remains associated with survival across different race and ethnic subgroups.^38^ We acknowledge there is some heterogeneity between the MESA and COPDGene cohorts and more so in the IPF cases. Another limitation is that the TOPMed and IPF cohorts were sequenced using different pipelines, such that residual between-cohort differences could not be fully harmonized. Although technical aspects were addressed within each cohort, absolute calibration of age-adjusted WGS-TL in the IPF cohort may still have been influenced by platform-specific differences. Thus, it is possible that the differences in WGS-TL between the IPF cases and participants without disease may be partly driven by technical variation. Subsequent validation in larger population and disease cohorts will be informative. Similar to other disease-oriented registries, the PFF-PR participants were required to have established disease at enrollment, so we were unable to quantify IPF development risk using WGS-TL. We did not have both WGS-TL and laboratory-based TL measurements in each PFF-PR patient, and thus could not directly compare their performance features at predicting TRQV status or survival. We used telomere gene QV as the “ground truth” by which to quantify the performance of our age-adjusted WGS-TL nomogram compared to raw WGS-TL values, as future studies should also compare against direct measurements from flow-FISH and qPCR. Since the confidence in qualifying pathogenicity of a novel variant can be highly variable, we also performed sensitivity analyses for protein-truncating variants and demonstrated largely the same results. Although we adjusted for potential confounders in our survival models, we cannot rule out residual confounding.^39-42^ Last, we used TelSeq to estimate TL, but other methods exist and it remains unclear if our approach to age adjustment will be reliable for those methods.

We present a scalable approach to age-adjust WGS-TL and demonstrate its superior diagnostic performance compared to raw WGS-TL. Age-adjusted WGS-TL may be a useful tool to identify individuals with abnormal telomere attrition and for qualifying telomere gene variants. Thus, incorporating age-adjusted WGS-TL estimation may enhance telomere gene variant discovery and enable broader application of large-scale WGS studies.

## Supplementary Material

aamag259_Supplementary_Data
