# Supplementary material for: Performance of age-adjusted whole genome sequencing telomere length in idiopathic pulmonary fibrosis
Source: Am J Respir Crit Care Med. 2026 Jun 8;212(10):2445–54. doi: 10.1093/ajrccm/aamag259 (PMC13624805; doi:10.1093/ajrccm/aamag259)
Supplement: Supplementary file 1 [file aamag259_supplementary_data.zip › 202511-274OC.Supplement.R1_2026-03-31.docx]

**Performance of Age-Adjusted Whole Genome Sequencing Telomere Length in Idiopathic Pulmonary Fibrosis**

**SUPPLEMENTARY APPENDIX**

**COPDGene Phase 3 Grant Support and Disclaimer**

The COPDGene study (NCT00608764) is supported by grants from the NHLBI (U01HL089897 and U01HL089856), by NIH contract 75N92023D00011, and by the COPD Foundation through contributions made to an Industry Advisory Committee that has included AstraZeneca, Bayer Pharmaceuticals, Boehringer-Ingelheim, Genentech, GlaxoSmithKline, Novartis, Pfizer and Sunovion. The content is solely the responsibility of the authors and does not necessarily represent the official views of the National Heart, Lung, and Blood Institute or the National Institutes of Health.

**COPDGene® Investigators – Core Units**

*Administrative Center*: James D. Crapo, MD (PI); Edwin K. Silverman, MD, PhD (PI); Barry J. Make, MD; Elizabeth A. Regan, MD, PhD

*Genetic Analysis Center*: Terri H. Beaty, PhD; Peter J. Castaldi, MD, MSc; Michael H. Cho, MD, MPH; Dawn L. DeMeo, MD, MPH; Adel El Boueiz, MD, MMSc; Marilyn G. Foreman, MD, MS; Auyon Ghosh, MD; Lystra P. Hayden, MD, MMSc; Craig P. Hersh, MD, MPH; Jacqueline Hetmanski, MS; Brian D. Hobbs, MD, MMSc; John E. Hokanson, MPH, PhD; Wonji Kim, PhD; Nan Laird, PhD; Christoph Lange, PhD; Sharon M. Lutz, PhD; Merry-Lynn McDonald, PhD; Dmitry Prokopenko, PhD; Matthew Moll, MD, MPH; Jarrett Morrow, PhD; Dandi Qiao, PhD; Elizabeth A. Regan, MD, PhD; Aabida Saferali, PhD; Phuwanat Sakornsakolpat, MD; Edwin K. Silverman, MD, PhD; Emily S. Wan, MD; Jeong Yun, MD, MPH

*Imaging Center*: Juan Pablo Centeno; Jean-Paul Charbonnier, PhD; Harvey O. Coxson, PhD; Craig J. Galban, PhD; MeiLan K. Han, MD, MS; Eric A. Hoffman, Stephen Humphries, PhD; Francine L. Jacobson, MD, MPH; Philip F. Judy, PhD; Ella A. Kazerooni, MD; Alex Kluiber; David A. Lynch, MB; Pietro Nardelli, PhD; John D. Newell, Jr., MD; Aleena Notary; Andrea Oh, MD; Elizabeth A. Regan, MD, PhD; James C. Ross, PhD; Raul San Jose Estepar, PhD; Joyce Schroeder, MD; Jered Sieren; Berend C. Stoel, PhD; Juerg Tschirren, PhD; Edwin Van Beek, MD, PhD; Bram van Ginneken, PhD; Eva van Rikxoort, PhD; Gonzalo Vegas Sanchez- Ferrero, PhD; Lucas Veitel; George R. Washko, MD; Carla G. Wilson, MS;

*PFT QA Center, Salt Lake City, UT*: Robert Jensen, PhD

*Data Coordinating Center and Biostatistics*, *National Jewish Health, Denver, CO*: Douglas Everett, PhD; Jim Crooks, PhD; Katherine Pratte, PhD; Matt Strand, PhD; Carla G. Wilson, MS

*Epidemiology Core*, *University of Colorado Anschutz Medical Campus, Aurora, CO*: John E. Hokanson, MPH, PhD; Erin Austin, PhD; Gregory Kinney, MPH, PhD; Sharon M. Lutz, PhD; Kendra A. Young, PhD

*Mortality Adjudication Core:* Surya P. Bhatt, MD; Jessica Bon, MD; Alejandro A. Diaz, MD, MPH; MeiLan K. Han, MD, MS; Barry Make, MD; Susan Murray, ScD; Elizabeth Regan, MD; Xavier Soler, MD; Carla G. Wilson, MS

*Biomarker Core*: Russell P. Bowler, MD, PhD; Katerina Kechris, PhD; Farnoush Banaei- Kashani, PhD

**COPDGene® Investigators – Clinical Centers**

*Ann Arbor VA:* Jeffrey L. Curtis, MD; Perry G. Pernicano, MD

*Baylor College of Medicine, Houston, TX*: Nicola Hanania, MD, MS; Mustafa Atik, MD; Aladin Boriek, PhD; Kalpatha Guntupalli, MD; Elizabeth Guy, MD; Amit Parulekar, MD;

*Brigham and Women’s Hospital, Boston, MA*: Dawn L. DeMeo, MD, MPH; Craig Hersh, MD, MPH; Francine L. Jacobson, MD, MPH; George Washko, MD

*Columbia University, New York, NY*: R. Graham Barr, MD, DrPH; John Austin, MD; Belinda D’Souza, MD; Byron Thomashow, MD

*Duke University Medical Center, Durham, NC*: Neil MacIntyre, Jr., MD; H. Page McAdams, MD; Lacey Washington, MD

*HealthPartners Research Institute, Minneapolis, MN*: Charlene McEvoy, MD, MPH; Joseph Tashjian, MD

*Johns Hopkins University, Baltimore, MD*: Robert Wise, MD; Robert Brown, MD; Nadia N. Hansel, MD, MPH; Karen Horton, MD; Allison Lambert, MD, MHS; Nirupama Putcha, MD, MHS

*Lundquist Institute for Biomedical Innovation at Harbor UCLA Medical Center, Torrance, CA*: Richard Casaburi, PhD, MD; Alessandra Adami, PhD; Matthew Budoff, MD; Hans Fischer, MD; Janos Porszasz, MD, PhD; Harry Rossiter, PhD; William Stringer, MD

*Michael E. DeBakey VAMC, Houston*, *TX*: Amir Sharafkhaneh, MD, PhD; Charlie Lan, DO *Minneapolis VA:* Christine Wendt, MD; Brian Bell, MD; Ken M. Kunisaki, MD, MS

*Morehouse School of Medicine, Atlanta, GA*: Eric L. Flenaugh, MD; Hirut Gebrekristos, PhD; Mario Ponce, MD; Silanath Terpenning, MD; Gloria Westney, MD, MS

*National Jewish Health, Denver, CO*: Russell Bowler, MD, PhD; David A. Lynch, MB *Reliant Medical Group, Worcester, MA*: Richard Rosiello, MD; David Pace, MD

*Temple University, Philadelphia, PA:* Gerard Criner, MD; David Ciccolella, MD; Francis Cordova, MD; Chandra Dass, MD; Gilbert D’Alonzo, DO; Parag Desai, MD; Michael Jacobs, PharmD; Steven Kelsen, MD, PhD; Victor Kim, MD; A. James Mamary, MD; Nathaniel Marchetti, DO; Aditi Satti, MD; Kartik Shenoy, MD; Robert M. Steiner, MD; Alex Swift, MD; Irene Swift, MD; Maria Elena Vega-Sanchez, MD

*University of Alabama, Birmingham, AL:* Mark Dransfield, MD; William Bailey, MD; Surya P. Bhatt, MD; Anand Iyer, MD; Hrudaya Nath, MD; J. Michael Wells, MD

*University of California, San Diego, CA*: Douglas Conrad, MD; Xavier Soler, MD, PhD; Andrew Yen, MD

*University of Iowa, Iowa City, IA*: Alejandro P. Comellas, MD; Karin F. Hoth, PhD; John Newell, Jr., MD; Brad Thompson, MD

*University of Michigan, Ann Arbor, MI:* MeiLan K. Han, MD MS; Ella Kazerooni, MD MS; Wassim Labaki, MD MS; Craig Galban, PhD; Dharshan Vummidi, MD

*University of Minnesota, Minneapolis, MN*: Joanne Billings, MD; Abbie Begnaud, MD; Tadashi Allen, MD

*University of Pittsburgh, Pittsburgh, PA*: Frank Sciurba, MD; Jessica Bon, MD; Divay Chandra, MD, MSc; Joel Weissfeld, MD, MPH

*University of Texas Health, San Antonio, San Antonio, TX*: Antonio Anzueto, MD; Sandra Adams, MD; Diego Maselli-Caceres, MD; Mario E. Ruiz, MD; Harjinder Singh

**Table E1**. PFF-PR IPF patient characteristics stratified by age-adjusted WGS-TL 10^th^ percentile

| **Characteristic** | **Above 10^th^ percentile (n=592)** | **Below 10^th^ percentile (n=233)** |
| --- | --- | --- |
| Age (years), mean (SD) | 72 (7) | 68 (8) |
| Female sex, n (%) | 135 (23) | 68 (29) |
| Self-reported race and ethnicity, n (%) |  |  |
| Non-Hispanic White | 534 (92) | 214 (96) |
| African American or Black | 5 (1) | 2 (1) |
| Hispanic | 23 (4) | 6 (3) |
| Asian | 18 (3) | 2 (1) |
| Ever smokers, n (%) | 385 (65) | 153 (66) |
| Cigarette pack-years, mean (SD) | 13.5 (15.6) | 13.2 (14.5) |
| Family history of pulmonary fibrosis, n (%) | 110 (19) | 63 (27) |
| FVC % predicted, mean (SD)* | 68 (17) | 68 (17) |
| DLCO % predicted, mean SD* | 41 (17) | 40 (13) |
| GAP stage, median (IQR)** | 5.0 (4.0-6.0) | 4.0 (3.0-5.0) |
| Pirfenidone exposure, n (%) | 139 (24) | 57 (24) |
| Nintedanib exposure, n (%) | 127 (22) | 50 (22) |
| Radiologic pattern, n (%)^*^ |  |  |
| Probable UIP | 91 (26) | 38 (26) |
| Definite UIP | 134 (38) | 53 (36) |
| Death or transplant, n (%) | 231 (39) | 100 (43) |
| Follow up time (months), median (IQR) | 36 (17-36) | 31 (16-36) |

*Excludes patients with missing FVC % predicted (n=34), DLCO % predicted (n=68)

**Excludes patients with either missing FVC % predicted for DLCO % predicted (n=70)

Acronyms: DLCO=diffusing capacity for carbon monoxide; FVC=forced vital capacity; GAP=gender-age-physiology; IQR=interquartile range; SD=standard deviation; UIP=usual interstitial pneumonia

**Table E2**. Distribution of raw WGS-TL across qualifying variants

| **Gene mutation** | **Raw WGS-TL Kb,**  **mean (SD)** | **Age-adjusted WGS-TL Kb,**  **mean (SD)** |
| --- | --- | --- |
| **Telomere-related rare variants** | **2.17 (0.51)** | **-0.99 (0.54)** |
| *TERT* (n=20) | 2.16 (0.65) | -1.05 (0.67) |
| *RTEL1* (n=15) | 2.11 (0.45) | -0.97 (0.41) |
| *PARN* (n=12) | 2.25 (0.37) | -0.98 (0.41) |
| *TERC* (n=9) | 2.13 (0.57) | -0.99 (0.70) |
| *NAF1* (n=2) | 2.33 (0.06) | -0.55 (0.09) |
| *TINF2* (n=1) | 2.19 (--) | -1.31 (--) |
| *ACD* (n=1) | 2.38 (--) | -0.81 (--) |
| **Non-telomere rare variants** | **3.33 (1.56)** | **0.23 (1.50)** |
| *ABCA3* (n=6) | 2.72 (0.99) | -0.37 (0.95) |
| *KIF15* (n=3) | 4.68 (2.14) | 1.55 (2.03) |
| *SPDL1* (n=1) | 2.95 (--) | -0.10 (--) |
| **Non-carrier (n=971)** | **2.63 (0.73)** | **-0.42 (0.74)** |

**Table E3**. Diagnostic performance characteristics identifying protein-truncating variants within telomere-related genes using age-adjusted and raw WGS-TL thresholds in PFF-PR IPF cohort (n=825)

|  | **Sensitivity** | **Specificity** | **PPV*** | **NPV*** | **AUC (95% CI)** |
| --- | --- | --- | --- | --- | --- |
| Family History | 0.39 | 0.80 | 0.06 | 0.97 | 0.59 (0.50-0.69) |
|  |  |  |  |  |  |
| Age-adjusted WGS-TL <50^th^ % | 0.96 | 0.20 | 0.04 | 0.99 | 0.58 (0.54-0.62) |
| Raw WGS-TL <50^th^ % | 0.68 | 0.45 | 0.04 | 0.98 | 0.56 (0.47-0.65) |
|  |  |  |  |  |  |
| Age-adjusted WGS-TL <10^th^ % | 0.54 | 0.73 | 0.06 | 0.98 | 0.63 (0.54-0.73) |
| Raw WGS-TL <10^th^ % | 0.29 | 0.89 | 0.08 | 0.97 | 0.59 (0.50-0.67) |
|  |  |  |  |  |  |
| Age-adjusted WGS-TL <1^st^ % | 0.14 | 0.99 | 0.31 | 0.97 | 0.57 (0.05-0.63) |
| Raw WGS-TL <1^st^ % | 0.07 | 0.99 | 0.22 | 0.97 | 0.53 (0.48-0.58) |

*Prevalence of telomere-gene qualifying variant = 7%

Abbreviations: AUC = area under the receiver operating characteristics curve; NPV = negative predictive value; PPV = positive predictive value; WGS-TL = whole genome sequencing estimated telomere length

**Table E4**. Diagnostic performance characteristics identifying qualifying variants within telomere-related genes using age-adjusted and raw WGS-TL thresholds in PFF-PR IPF cohort stratified by family history of pulmonary fibrosis (n=825)

|  | **Sensitivity** | **Specificity** | **PPV** | **NPV** | **AUC (95% CI)** |
| --- | --- | --- | --- | --- | --- |
| Familial Pulmonary Fibrosis* |  |  |  |  |  |
| Age-adjusted WGS-TL <50^th^ % | 1.0 | 0.22 | 0.18 | 1.0 | 0.61 (0.58-0.65) |
| Raw WGS-TL <50^th^ % | 0.92 | 0.47 | 0.23 | 0.97 | 0.69 (0.63-0.76) |
|  |  |  |  |  |  |
| Age-adjusted WGS-TL <10^th^ % | 0.80 | 0.70 | 0.32 | 0.95 | 0.75 (0.67-0.84) |
| Raw WGS-TL <10^th^ % | 0.48 | 0.89 | 0.41 | 0.91 | 0.68 (0.58-0.79) |
|  |  |  |  |  |  |
| Age-adjusted WGS-TL <1^st^ % | 0.24 | 0.99 | 0.86 | 0.89 | 0.62 (0.53-0.70) |
| Raw WGS-TL <1^st^ % | 0.20 | 1.0 | 1.0 | 0.88 | 0.60 (0.52-0.68) |
| Sporadic IPF** |  |  |  |  |  |
| Age-adjusted WGS-TL <50^th^ % | 0.97 | 0.20 | 0.06 | 0.99 | 0.59 (0.55-0.62) |
| Raw WGS-TL <50^th^ % | 0.69 | 0.46 | 0.07 | 0.96 | 0.57 (0.49-0.66) |
|  |  |  |  |  |  |
| Age-adjusted WGS-TL <10^th^ % | 0.43 | 0.75 | 0.09 | 0.96 | 0.59 (0.50-0.67) |
| Raw WGS-TL <10^th^ % | 0.26 | 0.90 | 0.13 | 0.96 | 0.58 (0.50-0.65) |
|  |  |  |  |  |  |
| Age-adjusted WGS-TL <1^st^ % | 0.06 | 0.99 | 0.33 | 0.95 | 0.53 (0.49-0.56) |
| Raw WGS-TL <1^st^ % | 0.03 | 1.0 | 0.25 | 0.95 | 0.51 (0.48-0.54) |

*Prevalence of telomere-gene qualifying variant = 14%

** Prevalence of telomere-gene qualifying variant = 5%

Abbreviations: AUC = area under the receiver operating characteristics curve; NPV = negative predictive value; PPV = positive predictive value; WGS-TL = whole genome sequencing estimated telomere length

**Table E5. Sensitivity analysis excluding patients with a family history of pulmonary fibrosis**

| **Group** | **Hazard ratio (95% CI)** |
| --- | --- |
| Exclude those with family history of pulmonary fibrosis (n=743) | 1.30 (1.01-1.67) |

HR reported among cases below age-adjusted 10^th^ percentile in comparison to those above 10^th^ percentile (reference group). ^*^ Cox regression model adjusted for GAP score, smoking history, and first 10 principal components of genetic ancestry.

**Table E6.** Stratified analyses by antifibrotic use and age for age-adjusted WGS-TL below 10^th^ percentile and transplant-free survival

| **Stratified Analysis** | **Hazard ratio (95% CI)** | **P-value for interaction** |
| --- | --- | --- |
| Antifibrotic use |  |  |
| No antifibrotic use at enrollment (n=405) | 1.48 (0.96-2.27) | 0.86 |
| Antifibrotic use at enrollment (n=350) | 1.25 (0.91-1.72) |  |
| Stratified by age |  | 0.04 |
| Age below 65 years (n=140) | 2.29 (1.30-4.03) |  |
| Age 65 years or older (n=615) | 1.14 (0.95-1.53) |  |

HR reported among cases below age-adjusted 10^th^ percentile in comparison to those above 10^th^ percentile (reference group). ^*^ Cox regression model adjusted for GAP score, smoking history, and first 10 principal components of genetic ancestry.

**Figure E1.** Locally estimated scatterplot smoothing (LOESS) plot of WGS-telomere length and chronological age in combined sample of COPDGene and MESA participants.

**Figure E2.** Distribution of raw WGS-Estimated Telomere Length across demographic and IPF-specific features

**Figure E3.** Distribution of age at enrollment for IPF patients in PFF-PR stratified by telomere-gene qualifying variants (A) and family history of pulmonary fibrosis (B).

**Figure E4.** Distribution of age-adjusted WGS-TL stratified by age groups within the PFF-PF IPF cohort (A) and restricted to IPF patients with a telomere-gene qualifying variant (B) and family history of PF (C). Distribution of raw WGS-TL stratified by age groups within the PFF-PF IPF cohort (D), restricted to IPF patients with a telomere-gene qualifying variant (E), and family history of PF (F).
